# An Unusual Presentation of a Mixed Epithelial and Stromal Tumor in an Elderly Male

**DOI:** 10.1100/tsw.2010.171

**Published:** 2010-09-14

**Authors:** Kelvin A. Moses, Irma V. Oliva, Adeboye O. Osunkoya, K. Jeff Carney

**Affiliations:** ^1^ Department of Urology, Emory University School of Medicine, Atlanta, GA, USA; ^2^ Department of Pathology and Laboratory Medicine, Emory University School of Medicine, Atlanta, GA, USA

**Keywords:** MEST, renal mass, cystic renal disease

## Abstract

Mixed epithelial and stromal tumors (MESTs) of the kidney are rare renal neoplasms characterized by mixed cystic and solid components. These tumors are typically present in middle-aged women as a flank mass, or as a cause of flank pain or hematuria. We outline the case of an older male who presented with an enlarging abdominal mass causing symptoms that suggested a partial small bowel obstruction. Management of the patient and a brief review are discussed.

## CASE REPORT

The patient is a 65-year-old Caucasian male who presented initially to an outside hospital with a complaint of abdominal pain, increasing abdominal girth, nausea and vomiting, and no flatus for 24 h. He had no associated hematuria, fevers, or weight loss. His past medical history was significant for coronary artery disease, status postcoronary artery bypass of three vessels, insulin-dependent diabetes mellitus on an insulin pump, chronic renal insufficiency with a baseline creatinine of 1.5 mg/dl, goiter, a left renal cyst, and nephrolithiasis. Medications included aspirin, furosemide, levothyroxine, and hydrocodone, and he had no allergies. His family history was significant for two brothers with known cystic renal disease. The patient is a retired veterinarian who is married, with a greater than 90 pack per year smoking history.

On physical examination, he was afebrile and tachycardic at 115 bpm. His blood pressure was 145/90, respirations were 22/min, and body mass index (BMI) was 33.4 kg/m$^2$. His abdomen was soft, significantly distended with left greater than right asymmetry, absent bowel sounds on the left, but no discrete point of tenderness. The remainder of his examination was normal, except for a neck mass consistent with his history of a goiter. Laboratory values included a potassium of 5.4 mEq/l, a creatinine of 3.9 mg/dl, a white blood cell count of 13.9 × 10$^3$/µl, urine culture positive for 20,000 cfu of *Enterococcus faecalis* and *Coryneform* spp, a C-reactive protein of 69.82 mg/l, and an estimated sedimentation rate of 41 mm/h. An MRI of the abdomen and pelvis revealed a 30.5- × 20.4-cm multiloculated or multiseptated cystic mass appearing to originate from the left kidney (Fig. 1a). This lesion had a significant mass effect on the adjacent small and large bowel without evidence of frank

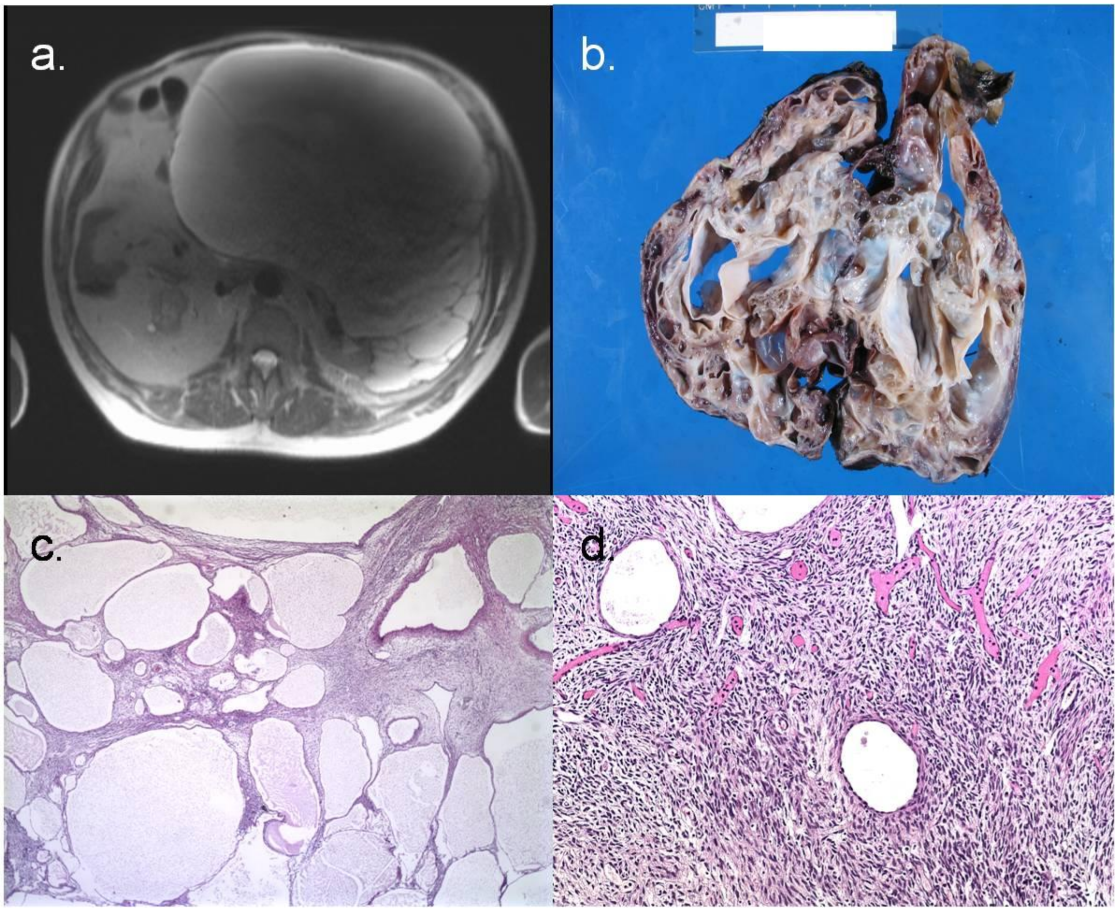

**FIGURE 1**. (a) MRI of left abdominal mass, (b) gross pathology specimen, (c) 2× microscopic section demonstrating multiloculated cysts with intervening stroma, (d) 10× microscopic section demonstrating variable cellularity, prominent vessels, and ovarian-type stroma.

obstruction. There were no enhancing septations or solid nodules with the mass. Due to the patient's significant pain and distention, as well as the low suspicion of malignancy, the mass was drained percutaneously. Cytology of the aspirated fluid showed inflammatory cells and histiocytes, but was negative for malignant cells.

Due to the patient's significant comorbidities, cardiac optimization was needed in the patient prior to surgical intervention and he required several drainage procedures due to reaccumulation of fluid. During this period, a MAG-3 study was obtained, demonstrating relative left-right renal function of 61 and 39%, with a clearance of 168 ml/min (expected 300 ml/min). Approximately 6 months after presentation, the patient was taken to the operating room, at which time he had cystoscopy, left retrograde pyelography, and exploratory laparotomy via a midline incision. There was a mass visualized in the left retroperitoneal cavity, which was removed in four sections. The mass eventually tapered to a stalk on the anterior surface of the kidney and a partial nephrectomy was performed.

Grossly, the tumor consisted of four sections weighing 1710 g, with the largest measuring 21 cm in greatest dimension (Fig. 1b). All fragments exhibited variable-sized cysts containing clear fluid. No normal renal parenchyma was identified. Microscopically, the mass showed a multiloculated tumor with a biphasic appearance. The epithelial component demonstrated a cystic pattern with individual cysts lined by benign cuboidal to hobnail epithelium. Intervening stroma showed variable cellularity and resembled ovarian-type stroma with prominent vessels and foci of hyalinization reminiscent of corpora albicans in the ovary (Figs. 1c and d).

Postoperatively, the patient had an ileus, which was managed conservatively, and a left abdominal fluid collection that was consistent with urine. This was managed with CT-guided drain placement. Serial imaging demonstrated resolution of the fluid collection over the next 4 weeks and the drain was removed without complication. At 2 years follow-up, the patient has done well, there has been no recurrence of the left renal mass, and his creatinine has stabilized at 1.2–1.4 mg/dl.

## DISCUSSION

Renal mixed epithelial and stromal tumor (MEST) is a benign entity that is frequently identified incidentally on imaging of the abdomen. These tumors are relatively rare and are usually described in case reports or small case series. MEST is a cystic lesion that may include solid areas and is biphasic, featuring both epithelial and stromal components. There is a female preponderance of patients with MEST in an approximate F:M ratio of 6:1[1]. MEST of the kidney is even more rarely described in men, and has been associated with estrogen therapy or hormonal therapy for prostate cancer[2,3]. Because of these findings, it has been suggested that the hormonal environment plays a role in the development of these tumors[1].

This case is unique in that it was identified in an older male with no history of estrogen exposure or treatment for prostate cancer. Additionally, the presentation of symptoms suggesting partial small bowel obstruction and regrowth of the mass secondary to fluid accumulation was quite unusual. Aspiration of cystic renal masses is not the standard treatment modality and, indeed, can place patients with malignant neoplasms at risk for tract seeding. However, there was low suspicion of malignancy, and due to the patient’s significant pain and severe comorbidities, we felt safe in proceeding with cyst aspiration. Finally, surgical planning allowed for a flexible approach, including the use of nephron-sparing surgery. The use of nephron-sparing surgery has been shown to preserve overall renal function and have an impact on survival, yet is underutilized as a surgical approach[4,5]. The ability to perform nephron-sparing surgery in the setting of such a large tumor was quite beneficial for this patient, as evidenced by the maintenance of his renal function.

The differential diagnosis of cystic renal masses and renal MEST includes both benign and malignant entities, and is guided by both microscopic and immunohistochemical features unique to various tumors. The most common differential is cystic nephroma (CN). It has been suggested that CN and MEST represent ends of a spectrum of cystic renal diseases, and similarities include both their behavior and morphology[2,6]. CN includes cystic regions, but does not usually have solid components. CN differs from MEST in that CN tumors are more likely to have larger cysts, thinner septa, and a lower stromal-to-epithelial ratio[6,7]. Recently, some have suggested that MEST and CN be included under the same umbrella term “renal epithelial and stromal tumor” (or REST). This is because both entities have the following similarities: (1) they are cystic masses that generally behave in a benign manner; (2) both are predominantly seen in middle-aged women; (3) they demonstrate cystic architecture, eosinophilic and hobnail cells, and ovarian stroma; and (4) both are immunohistochemically similar, expressing estrogen and progesterone receptor positivity, CD10, calretinin, and inhibin[2,7].

Other diagnoses to be considered include congenital mesoblastic nephroma (CMN), multicystic clear cell renal cell carcinoma (CC-RCC), and renal synovial carcinoma. CMN is distinct from MEST in that it usually presents as a solid mass with an infiltrating interface with the renal parenchyma, whereas MEST is usually well circumscribed. Additionally, CMN exhibits genetic alterations that are not seen in MEST[3]. Multicystic CC-RCC differs from MEST in that it contains aggregates of clear cells[1,3]. Renal synovial sarcoma is a more aggressive tumor that has some similarities to malignant MEST. However, expression of the STY-SSX2 transcript and the lower likelihood of ovarian-type stroma sets renal synovial sarcoma apart from MEST[3,8].
